# Efficacy of bortezomib combined with Hyper-CVAD in adults with relapsed acute lymphoblastic leukemia or positive measurable residual disease; effect of bortezomib in leukemia

**DOI:** 10.1007/s44313-024-00050-6

**Published:** 2025-01-14

**Authors:** Christian Omar Ramos Peñafiel, Daniela Pérez Sámano, Adán Germán Gallardo Rodríguez, Camila Terreros Palacio, Irma Olarte Carrillo, Carlos Martínez Murillo, Gilberto Barranco Lampón, Álvaro Cabrera García, Adolfo Martínez Tovar

**Affiliations:** 1 Hematology Department, General Hospital of Mexico “Dr. Eduardo Liceaga, Mexico City, Mexico; 2 Hematology Department, Regional Hospital of High Specialty of Ixtapaluca, State of Mexico Mexico City, Mexico; 3 Hematology Research Department, General Hospital of Mexico “Dr. Eduardo Liceaga”, Mexico City, Mexico; 4 Hematology Laboratory, General Hospital of Mexico “Dr. Eduardo Liceaga”, Mexico City, Mexico

**Keywords:** Bortezomib, Chemotherapy, Acute Lymphoblastic Leukemia, Relapse, Measurable Residual Disease

## Abstract

**Purpose:**

Despite advances in the treatment of adult acute lymphoblastic leukemia (ALL), relapse remains the most significant challenge in improving prognosis. Measurable residual disease (MRD) assessment can predict bone marrow relapse based on MRD positivity. As access to innovative therapies remains limited because of the high cost, chemotherapy is the widely utilized treatment option. The efficacy of a combination of bortezomib and Hyper-CVAD has been reported in patients with multiple myeloma; however, its efficacy has not yet been confirmed in patients with ALL.

**Methods:**

This prospective cohort study involved patients with ALL who presented with MRD-positive results or relapse and received treatment with a combination of bortezomib and Hyper-CVAD at two reference centers in Mexico City.

**Results:**

Of the 20 patients with positive MRD included in this study, 60% (*n* = 12) exhibited MRD negative results after combination treatment, 30% (*n* = 6) persisted positive MRD results, and 10% (*n* = 2) passed away. Of the 23 patients with bone marrow relapse, 43.5% (*n* = 10) achieved a second complete remission (2CR), 34.8% (*n* = 6) exhibited refractory status, and 21.7% (*n* = 5) passed away. To achieve a 2CR, 20% (*n* = 2) patients required less than four cycles of treatment, 50% (*n* = 5) required four cycles (two A and B cycles each), and 30% (*n* = 3) required six cycles.

**Conclusion:**

The combination of bortezomib and Hyper-CVAD treatment exhibited better results in achieving MRD negative results, indicating its potential as a promising first-line treatment strategy for ALL.

## Introduction

Despite advances in the treatment of acute lymphoblastic leukemia (ALL) in adults, relapse, particularly during the first months after diagnosis, is the most significant challenge in improving patient prognosis. Although the presence of the Philadelphia chromosome T (9,22: q34: q11) and *KMT2A* alterations indicate worst prognosis, measurable residual disease (MRD) positivity remains the primary prognostic factor during treatment [1–3].

MRD is determined using flow cytometry, and positive MRD results predict the occurrence of bone marrow relapse [4]. Currently, blinatumomab—a bispecific monoclonal antibody targeting CD19 and CD3 to induce direct cytotoxicity—is the only beneficial strategy for patients with MRD positivity [5]. Blinatumomab treatment in MRD-positive cases can neutralize the disease by up to 78% [6], presenting it as a notable option before hematopoietic stem cell transplantation. Similar results have been observed in relapse cases, where blinatumomab treatment improves outcomes compared with the standard chemotherapy (disease-free survival of 31% vs. 12%) [7].

However, access to these innovative therapies is limited, particularly in Latin America, owing to their high cost. Chemotherapy remains the most common treatment option for patients with relapse, including a combination of various regimens involving purine analogs (FLAG and FLAD-Ida), chemotherapy different from the induction regimen (mitoxantrone and etoposide), or an increase in the dosage of the standard regimen (augmented Hyper-CVAD) [8, 9].

The selection of each regimen depends on the experience of the treatment center and the tolerance of each patient to higher-intensity therapies. Besides chemotherapy, a limited number of drugs are used for ALL treatment in relapse cases. Bortezomib is a first-generation proteasome inhibitor widely used to treat multiple myeloma dyscrasia and mantle cell lymphoma [10, 11]. Notably, bortezomib has been reported to halt the cell cycle, arresting cells in the G1 phase and inducing apoptosis in leukemia cell lines (MOLT-4) [12]. This effect is not exclusive to bortezomib, and carfilzomib, an irreversible proteasome inhibitor, has demonstrated synergy with drugs used to treat ALL [13]. Bortezomib is also used for treating pediatric patients, and improved results have been obtained for specific groups, such as those with T variants, when combined with reinduction chemotherapy (vincristine, prednisone, pegylated asparaginase, doxorubicin [AALL01P2]) [14]. The combination of bortezomib with more aggressive regimens (cyclophosphamide and liposomal doxorubicin) has been previously explored in patients with multiple myeloma without an increase in toxicity. However, the effect of this combination in adults with ALL remains insufficiently explored [15].

As MRD positivity after induction is the leading risk factor for relapse, this study aimed to evaluate the effect of bortezomib addition to an intensified hyper-CVAD regimen to neutralize MRD and increase remission rate in relapse cases [16].

## Materials and methods

This prospective cohort study involved patients diagnosed with ALL type B treated at the Hospital General de México “Dr. Eduardo Liceaga” and the Hospital Regional de Alta Especialidad de Ixtapaluca. The inclusion criteria were as follows: (1) both sexes, (2) age > 18 years, (3) receiving systemic chemotherapy treatment, and (4) occurrence of a bone marrow relapse (> 10% of blasts) or MRD positivity confirmed using multiparametric flow cytometry (> 0.01) or high-risk ALL at diagnosis. Patients (1) having ECOG score > 2 or severe asparaginase toxicity, (3) diagnosed with biphenotypic leukemia, (4) receiving palliative care or transfusion support, (5) exhibiting severe comorbidities that could jeopardize the therapy, and (6) a history of cardiac toxicity or arrhythmias associated with the treatment were excluded.

The standard treatment regimen used at both centers was the CALGB 10403 protocol, which has been adopted as the reference chemotherapy regimen for adult patients with ALL in Mexico. This protocol was selected based on its efficacy and safety profile in the patient population, providing a uniform therapeutic approach across the study cohort.

### Procedure

The patients meeting the inclusion criteria were followed up for two cycles of chemotherapy with Hyper-CVAD plus bortezomib. Follow-ups were performed during the hospital stay, and clinical and biochemical indicators and prognostic factors of the patients were monitored.

The final MRD test was performed using samples obtained from the bone marrow. Briefly, 5 mL blood was extracted from the bone marrow, added to EDTA tubes, and analyzed via flow cytometry using the markers for CD19, CD10, and CD34 + expression on the lymphoid blasts.

Complete remission was considered when patients presented with < 5% blasts in the bone marrow after the treatment cycle. The time to remission and the number of cycles required to achieve remission were analyzed.

### Chemotherapy treatment

The chemotherapy regimen was divided into two blocks: the first, denoted as cycle A, involved cyclophosphamide (300 mg/m^2^ per body surface area) administration every 12 h on days 1, 2, and 3, along with vincristine (1.2 mg/m^2^ per body surface area) on days 1, 8, and 15 of the treatment. Furthermore, doxorubicin was administered at a dose of 50 mg/m^2^ per body surface area on day + 4, and pegylated asparaginase was substituted with *Escherichia coli*-synthesized asparaginase at a dose of 5000 UI /m^2^ per body surface area administered as six doses at day + 5 of the chemotherapy. Rituximab was administered in conjunction with each treatment cycle in patients with CD20 + ALL, whereas a tyrosine kinase inhibitor (imatinib or dasatinib) was added to the chemotherapy regimen for patients expressing BCR-ABL1.

Bortezomib was administered subcutaneously at a dose of 1.3 mg/m^2^ per body surface area on days + 1, + 4, + 8, and + 11 of chemotherapy. During cycle B, high doses of methotrexate (1.5 g/m^2^ per body surface area) were administered via continuous infusion on day + 1 of the cycle, followed by cytarabine (1 g/m^2^ per body surface area) every 12 h on days + 2 and + 3 of the treatment. Bortezomib was administered in the same manner during treatment cycles A and B.

CNS prophylaxis was administered via intrathecal chemotherapy. Specifically, 15 mg intrathecal methotrexate was administered on day + 2, followed by 100 mg intrathecal cytarabine on day + 8 of each treatment cycle. This prophylactic regimen was implemented to reduce the risk of central nervous system relapse and was maintained throughout the treatment period.

The toxicity of the regimen was evaluated according to the NCI Common Toxicity Criteria for Adverse Events version 4.016; severe toxicity was considered for grade 4 cases. In cases of severe toxicity (grade 3 or grade 4) associated with bortezomib, administration was halted for subsequent cycles, and patients treated with bortezomib were administered antifungal and antiviral prophylaxis.

### Ethical disclosures

All patients provided written informed consent for the study and data collection. The study procedures were performed following the Declaration of Helsinki guidelines and approved by the Biosecurity, Ethics and Research Committee of Hospital General de México “Dr. Eduardo Liceaga” (protocol number: HGMDI/21/204/03/67). The study was also registered at ClinicalTrial.gov (registration code: NCT05137860).

### Statistical analysis

To describe the demographic variables, the Shapiro–Wilk test was used to estimate the normality distribution of the numeric variables. The proportions of principal risk factors were evaluated in patients with relapse or MRD positivity. Furthermore, an analysis of survival (Kaplan–Meier analysis) and a log-rank test on overall survival were performed in both groups. Statistical analyses were performed using Med-Calc 20.009 (New York, NY C.P. 10,003, USA) and SPSS version 25 (IBM, Armonk, NY, USA), and figures were generated using GraphPad Prism version 7. Statistical significance was set at *p* < 0.05.

## Results

### General characteristics of the patients

Forty-three patients (51.2% [n = 22] males and 48% [*n* = 21] females) with bone marrow relapse or MRD positivity at the end of induction were analyzed. Of these, 46.6% (*n* = 20) and 53.5% (*n* = 23) exhibited MRD positivity and bone marrow relapse, respectively. In terms of diagnosis, most patients (95.3%) exhibited B-cell phenotype, 14% (*n* = 6) presented central nervous system involvement at diagnosis, and 7% (*n* = 3) exhibited Philadelphia chromosome. Overall, 86% of the cohort was considered high-risk. Regarding initial response to treatment, 65.1% (*n* = 28) patients achieved complete remission within 4 weeks, whereas 34.9% (*n* = 15) exhibited refractory status to the first treatment regimen. Analysis of the MRD status after induction of patients who achieved complete remission revealed MRD-negative and -positive results for 41.9% (*n* = 18) and 44.2% (*n* = 19) patients, with 14% (*n* = 6) with unavailable MRD results.

Among the patients with the T-cell phenotype, 100% achieved a second complete remission (2CR) following the first cycle of augmented Hyper-CVAD plus bortezomib. The demographic characteristics of the patients are presented in Table 1.

**Table 1:** Demographic characteristics of the patients

	MRD-positive (***n*** = 20)	Relapse (***n*** = 23)
Age (years)	26.35 (18–40)	27.73 (18–58)
Gender (M:F)	11:9	10:13
WBC count (× 10^3^) at diagnosis	32.00 (1.50–253.00)	27.90 (0.30–414.20)
Philadelphia chromosome		
Absence	18 (90.0%)	22 (95.7%)
Presence	2 (10.0%)	1 (4.3%)
Immunophenotype		
B cell precursor ALL	20 (100.0%)	21 (91.3%)
T cell precursor ALL	0 ( 0.0%)	2 (8.7%)
CNS infiltration at diagnosis		
Negative	19 (95.0%)	18 (78.3%)
Positive	1 (5.0%)	5 (21.7%)
Risk at diagnosis		
Standard	4 (20.0%)	2 (8.7%)
High	16 (80.0%)	21 (91.3%)

*M* Male, *F* Female, *WBC* White Blood-cell, *CNS* central nervous system. The values are expressed as means (rates) for the quantitative variables and absolute values (%) for the qualitative variables

### Treatment response

#### Response in MRD-positive cases

Responses in 20 patients with MRD positivity treated with augmented Hyper-CVAD and bortezomib combination were analyzed. Of these, 60% (*n* = 12) patients exhibited neutralization following treatment, 30% (*n* = 6) exhibited positive results, and 10% (*n* = 2) passed away during intensification.

Of the patients with MRD neutralization, 25% (*n* = 3) required completion of cycles A and B and 66.6% (*n* = 8) achieved neutralization after three or more treatment cycles (a maximum of four cycles). Both deaths occurred during the first two treatment cycles (cycles A and B).

Maximum four treatment cycles were administered in this study, as established in the treatment protocol. This limit was set to balance the therapeutic efficacy and minimize treatment-related toxicity. Patients who did not achieve MRD negativity after four cycles were considered non-responders and evaluated for alternative therapeutic strategies. Treatment response characteristics of the patients are presented in Table 2.

**Table 2:** Treatment response characteristics of the patients

	**MRD-positive (*n* = 20)**	**Relapse (*n* = 23)**
Treatment response		
Favorable	12 (60.0%)	10 (43.5%)
Refractory	6 (30.0%)	8 (34.8%)
Death	2 (10.0%)	5 (21.7%)
Treatment response ALL-Ph +		
Favorable	1 (50.0%)	1 (100%)
Refractory	1 (50.0%)	(0.0%)
Death	0 (0.0%)	(0.0%)
Overall Survival		
Alive	16 (80.0%)	4 (17.4%)
Death	4 (20.0%)	19 (82.6%)
Overall survival (days)	537 (187–1000)	648 (168–1000)

The values are expressed as medians (rates) for the quantitative variables and absolute values (%) for the qualitative variables

#### Results in relapse cases

Of the 23 patients with bone marrow relapse, 43.5% (*n* = 10) achieved 2CR, 34.8% (*n* = 6) exhibited refractory status, and 21.7% (*n* = 5) passed away during the rescue therapy. In particular, 20% (*n* = 2) patients achieved 2CR in less than four cycles, 50% (*n* = 5) required four treatment cycles (two A and B cycles each), and 30% (*n* = 3) required six cycles. Notably, 60% (*n* = 3) of the deaths occurred during the first cycle, whereas the rest (40%, *n* = 2) occurred between cycles two and three.

### Toxicity and outcome

All 43 patients who received treatment experienced grade 4 hematological toxicity requiring granulocyte colony-stimulating factor and transfusion support. Febrile neutropenia occurred in 62.8% (n = 27) patients, necessitating broad-spectrum antibiotic treatment. Herpetic viral infection was observed in 7% (n = 3) of patients who required antiviral therapy.

No severe neurotoxicity related to the use of proteasome inhibitors was reported, and only 13.9% of patients experienced mild dysesthesia and paresthesia (grade 1). Bortezomib was not concurrently administered with vincristine to minimize the risk of neurotoxicity.

Regarding the causes of death among patients with MRD positivity, four deaths occurred during treatment. In contrast, 19 (82.6%) patients with bone marrow relapse succumbed to the disease. Notably, 20 deaths were attributed to sepsis associated with febrile neutropenia, whereas the remaining deaths were associated with disease progression.

Regarding the management of patients with MRD positivity, of the six patients with persistent MRD positivity following treatment with augmented Hyper-CVAD plus bortezomib, three achieved MRD negativity after receiving blinatumomab as salvage therapy. Additionally, two of these patients were eligible for hematopoietic stem cell transplantation and successfully underwent the procedure. In contrast, among patients with relapse, only three could undergo transplantation, as most of them had comorbidities or lacked a suitable donor, limiting this therapeutic option.

### Overall survival

**Fig. 1:**
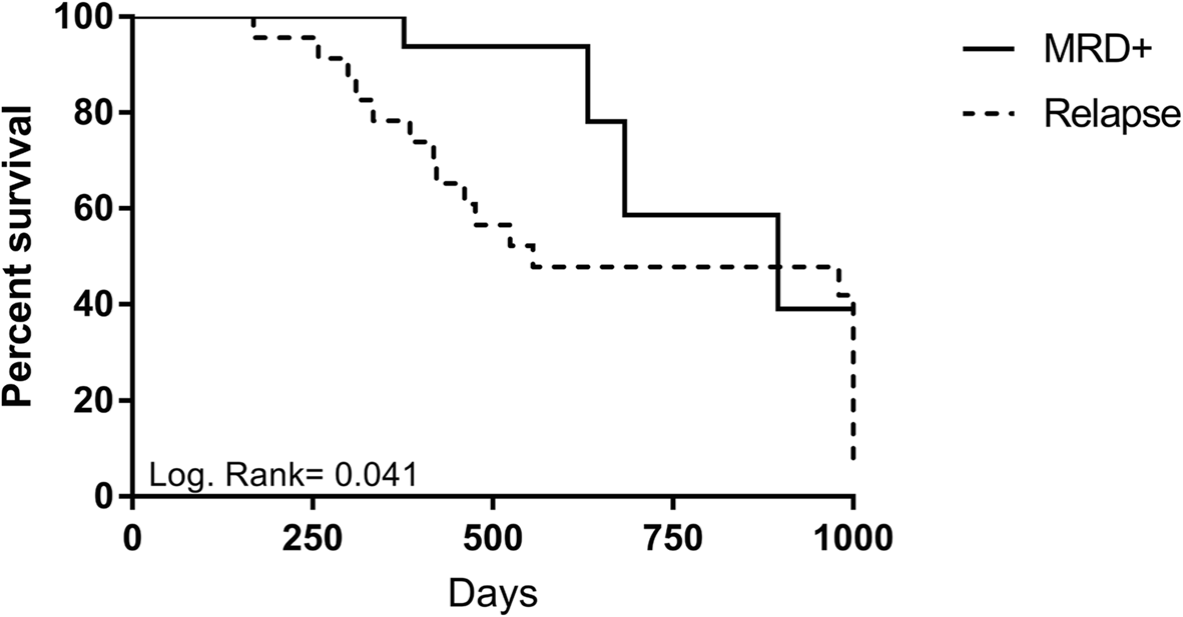
Survival rate among patients with MRD positivity and relapse

**Fig. 2:**
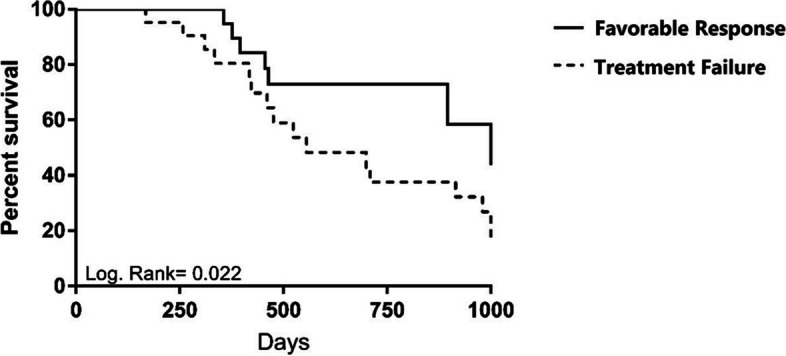
Survival rate among patients according to the response to the combination treatment with Hyper-CVAD and bortezomib

The average survival rate was 596 days (168–1000 days). Survival was higher in patients with MRD positivity (Fig. 1), as well as in those considered to have achieved a favorable response (Fig. 2).

## Discussion

Despite the progress and integration of immunotherapy into ALL treatment in adults, the prognosis remains unfavorable in most centers with no access to a hematopoietic progenitor cell transplantation unit. Furthermore, the toxicity of rescue regimens and the risk of opportunistic infections remain major limitations in developing countries [17–19]. The rescue regimens more frequently involve a purine analog, and other regimens employ higher doses of chemotherapy to overcome resistance associated with treatment [20]. This study evaluated the efficacy of bortezomib, a first-generation proteasome inhibitor, in combination with the augmented Hyper-CVAD regimen, owing to its demonstrated in vitro synergy for the treatment of patients with relapse and MRD positivity.

In relapse cases, the rate of 2CR was 43.5%, comparable to the regimens involving fludarabine alone or in combination with idarubicin (33.3% to 67%) [8, 21–24], as well as those using clofarabine (32–45%), which are better tolerated but require a higher number of cycles to achieve 2CR [25, 26].

The remission rate observed in this study was slightly lower than that reported for the original augmented Hyper-CVAD regimen (43.5% vs. 47%) [27]; however, it is comparable to other regimens based exclusively on chemotherapy (mitoxantrone and etoposide) [28, 29]. Although a more significant benefit for patients with relapse was not identified, the combination treatment demonstrated benefits for patients with MRD positivity, achieving a 60% neutralization rate, albeit requiring several cycles for the effect to become evident.

Before the era of immunotherapy, positive MRD was considered the only prognostic factor for relapse [30, 31]. In Latin America, Ferrari et al. reproduced the impact of MRD positivity (on days 33 and 78) on the survivability free of relapse (HR 3.0, 95% CI 1.6–5.7 and HR 2.6, 95% CI 1.3–5.1) in young patients treated with the adapted BFM ALL IC 2009 protocol [32]. Currently, blinatumomab is the preferred treatment choice for patients with MRD positivity, with a 78% neutralization rate following one cycle [6, 33].

These advances have not yet been reflected in Latin America because of the limited access to the MRD assessment using molecular techniques, drugs such as blinatumomab, and hematopoietic stem cell transplantation units [34], necessitating the validation of other combinations, such as those involving proteasome inhibitors.

Bortezomib is considered an attractive drug for combination owing to its synergistic effect with most drugs used in ALL treatment through its effects on nuclear factor kappa B [35]. To date, its most significant benefit has been demonstrated in pediatric patients with precursor T-cell leukemia when used in combination with other drugs, such as ruxolitinib or venetoclax [36]. Different combinations explored in this population include mitoxantrone, vincristine, and PEG-asparaginase, achieving complete remission in 80% cases [37]. Although most of this data is based on pediatric trials, Nachmias et al. evaluated the effect of bortezomib in nine adult patients (five patients with ALL type B) and presented favorable responses in seven patients without any severe adverse events [38]. As most of the previous trials involved patients with relapse or refractory (R/R) status, we sought to evaluate the efficacy of the combination treatment in neutralizing MRD. Negative MRD results were achieved in 60% cases, requiring at least three cycles of treatment, in contrast to outcomes observed in the relapse cases. Similarly, Jonas et al. evaluated a similar combination (Hyper-CVAD) in a Phase I trial and achieved 80% MRD-negative results without cardiovascular events using carfilzomib, a second-generation inhibitor [39]. In contrast, Jain et al. evaluated the synergy of bortezomib with rituximab in patients aged > 14 years with de novo ALL and reported 70.9% MRD negativity post-induction with improved disease-free survivability [40].

In conclusion, bortezomib may be considered when using an augmented Hyper-CVAD therapy, with MRD neutralization being its most significant benefit. Following further investigations through trials, bortezomib can be incorporated into first-line regimens for adults with ALL.

## Data Availability

Due to privacy and confidentiality restrictions, the data presented in this study is available on request from the corresponding author. The data are not publicly available due to the confidentiality restrictions of the Biosecurity, Ethics, and Research Committee of Hospital General de México “Dr. Eduardo Liceaga”.
